# Distinguishing Among Causes of Death for Patients with Kidney Failure on Hemodialysis

**DOI:** 10.34067/KID.0000000681

**Published:** 2024-12-16

**Authors:** Michelle Tran, Chun Anna Xu, Jonathan Wilson, Patti L. Ephraim, Tariq Shafi, Daniel E. Weiner, Benjamin A. Goldstein, Julia J. Scialla

**Affiliations:** 1 Department of Medicine, University of Virginia School of Medicine, Charlottesville, Virginia; 2 Department of Biostatistics and Bioinformatics, Duke University School of Medicine, Durham, North Carolina; 3 Institute of Health System Science, Feinstein Institute for Medical Research, Northwell Health, New York, New York; 4 Division of Nephrology, Department of Medicine, Houston Methodist Hospital, Houston, Texas; 5 Department of Medicine, Tufts Medical Center, Boston, Massachusetts; 6 Department of Public Health Sciences, University of Virginia School of Medicine, Charlottesville, Virginia

**Keywords:** cardiovascular disease, chronic dialysis, dialysis, epidemiology and outcomes, kidney failure, mortality, survival, prediction modeling

## Abstract

**Key Points:**

- We found poor ability to distinguish among different causes of death using clinical information in the 30 days before death for patients on hemodialysis.
- We found that models of different causes of death identified similar associated clinical factors.
- Given the lack of clear phenotypic differences, this study questions the usefulness of cause of death in research in dialysis.

**Background:**

Patients treated with maintenance hemodialysis are at high risk of death from a variety of causes.

**Methods:**

To identify markers (*i.e*., risk phenotypes) that distinguish among causes of death, we used dialysis electronic health record data for a cohort of adults treated with maintenance in-center hemodialysis who died between 2003 and 2016 (*n*=19,793). Patients were linked to the United States Renal Data System (USRDS) Files. We classified USRDS-reported causes of death into five categories: sudden cardiac death, nonsudden cardiac death cardiovascular death, infection, others, and unknown. A subcohort was linked to the National Death Index with similar categories defined. We used ensemble classification trees to discriminate among causes using demographics, vital signs, laboratory measures, health service utilization, and comorbidity claims from 30 days before death. We then created nested case-control populations for each cause of death and used ridge logistic regression to evaluate clinical risk markers that associate with distinct causes.

**Results:**

The area under the receiver operating characteristic curves from ensemble classification trees were all between 0.59 and 0.70, suggesting minimal ability to distinguish among causes using clinical risk markers. Model coefficients were similar and highly correlated across different cause of death models (*i.e*., 0.87–0.94). This suggests that most clinical risk markers are shared across causes without distinct risk phenotypes.

**Conclusions:**

We conclude that different causes of death may share similar clinical risk markers in the setting of kidney failure or that the causes of death attributed on USRDS or National Death Index forms are not precise.

## Visual Abstract

**Figure:**
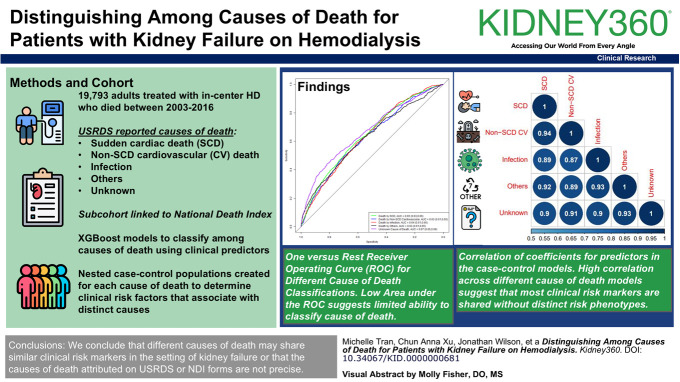

## Introduction

Patients treated with maintenance hemodialysis are at high risk of death, with an estimated 1-year mortality rate of approximately 20%.^1^ The drivers of high mortality are largely presumed to be cardiovascular disease and infection.^1–5^ However, other causes of death, including malignancy, malnutrition/cachexia, and others, are increasingly recognized in epidemiologic studies and should be carefully considered.^6,7^ Furthermore, among dialysis patients, nonatherosclerotic cardiovascular events, including heart failure and sudden death, are disproportionately common compared with the general population.^8,9^

Distinguishing among different causes of death may promote individualized management focused on the particular patient risk phenotype. For instance, patients with an atherosclerotic cardiovascular risk phenotype may be worsened by intradialytic hypotension and associated myocardial ischemia. In this instance, risk could be addressed with dietary sodium restriction and longer or more frequent hemodialysis sessions to reduce interdialytic weight gain and ultrafiltration needs.^10^ Patients with a risk phenotype for sudden death might consider dietary potassium restriction and potassium binders, changes in dialysate prescriptions, and careful review of QTc prolonging medications.^11–13^ Individuals with a risk phenotype related to other causes, such as malnutrition/cachexia, may be prescribed a more liberal diet.^14^ The current care model for patients on hemodialysis uses standardized protocols and biometric targets. Improving our understanding of distinct clinical risk phenotypes may promote more customization of care.

The dialysis environment is potentially well suited to accurate classification of the cause of death. Patients with kidney failure receiving in-center hemodialysis are seen regularly by medical professionals, yielding multiple opportunities for close clinical assessment. Extensive laboratory, biometric, and medication data are regularly collected at frequent intervals. Clinicians at dialysis facilities, who have regular contact with patients and knowledge of their health status, are required to classify cause of death through mandated reporting on the US Centers for Medicare & Medicaid Services (CMS) Form 2746.^1^ For these reasons, we hypothesized that recent clinical information available in the dialysis electronic health records (EHRs) may be able to distinguish among different causes of death, identifying meaningfully distinct risk phenotypes.

In this study, we use detailed EHR data from patients treated with maintenance in-center hemodialysis at facilities affiliated with Dialysis Clinic, Inc. (DCI). Our goal is to assess whether it is feasible to use routinely collected health information to classify individuals who died into distinct causes of death. This may identify differentiating factors that make up definable risk phenotypes.

## Methods

### Data

#### Data Sources and Study Population

We used three data sources for this analysis. Our primary data source consisted of EHR data from patients with kidney failure who began receiving maintenance in-center hemodialysis at a facility affiliated with DCI between 2003 and 2016. We linked these data with the United States Renal Data System (USRDS) standard analytic files covering the same period. We used the USRDS data to identify mortality and claims-based comorbidities. In a sensitivity analysis, we also include linked data from the National Death Index (NDI) available on a subcohort of patients between 2003 and 2009.^15^ This study was approved by the Duke University Institutional Review Board under Pro# 00105834, along with a waiver of informed consent.

Because our goal was to focus our analyses on features that may distinguish among causes of death, we selected all adult patients in our DCI cohort who died between 2003 and 2016 and who had actively received treatment at a DCI facility within 30 days of death. All patients were required to remain on dialysis therapy at least 90 days after the first dialysis service date to be included. We excluded patients with a previous failed kidney transplant.

#### Outcomes

We used the USRDS death file sourced from CMS Form 2746. This provides cause of death as indicated by the dialysis physician. There are 77 unique causes of death. We categorized these into five categories: sudden cardiac death (SCD), non-SCD cardiovascular death, infection, others, and unknown (see Supplemental Table 1). We used an assignment scheme adapted from the approach used in the USRDS Annual Data Report.^1,16^ Some groups were further combined to ensure each group had an adequate sample size for the classification task.

In a sensitivity analysis, for patients who died between 2003 and 2009, we used the NDI for final cause or condition that resulted in death. The NDI information is derived from the official death certificate completed either by medical examiners or coroners. The death certificate is frequently completed by primary care physicians or inpatient physicians rather than the outpatient nephrologist. We extracted data from the first entity condition supplied in the cause of death section of the death certificate, presented in the form of ICD-10 codes. We mapped the cause of death to the same five categories as USRDS outcomes (see Supplemental Table 2), similar to our prior report.^16^

#### Predictor Variables

We extracted the most recent information from the DCI EHR from the 30 days before an individual died. Variables included demographics, vital signs, laboratory measures, health service utilization, and comorbidity claims (see Supplemental Table 3 for variable definitions). We calculated the calendar month-wide average for variables measured more than once in a month (*e.g*., pre-dialysis systolic BP). We used last observation carried forward for variables not measured in the last 30 days. We defined plausible values and removed any measurements outside that range (see Supplemental Table 4 for degree of missingness). In total, we had 53 predictor variables.

#### Comparative Controls

In secondary analyses, we evaluated clinical factors that may differentially associate with different causes of death using a nested case-control design, temporally matched. For each patient who died of a specific cause, we identified a relevant control patient. Using the calendar month of the patient’s death, we randomly sampled a living eligible patient who was still alive and active in DCI in the previous calendar month. This resulted in unique control groups for each cause of death. Aside from an encounter at the same point in time, no other restrictions were used. Of note, patients were eligible to serve as both a case and control at different times, as is appropriate in a nested case-control design.^17^

### Analytic Approach

We first describe the eligible cohort based on their demographic characteristics, stratifying on cause of death. We report standardized mean differences (SMDs) to provide indications of how individuals differ.

#### Classifying Cause of Death

We divided the data into training (75%) and testing sets (25%). We fit a multinomial classification model using Extreme Gradient Boosting (XgBoost) to classify each of the five potential cause of death groups.^18^ This produces, for each person, a set of probabilities for each cause of death that sums to one. XgBoost is a machine learning, tree-based algorithm that can flexibly model complex (*i.e*., nonlinear and heterogeneous) relationships. We note that, in sensitivity analyses, results were similar when using a simpler least absolute shrinkage and selection operator (LASSO) regression-based approach (results not shown). We used ten-fold cross-validation on the training data to choose the optimal XgBoost hyperparameters.

We applied the model to the test data to assess fit. We first calculated cross-entropy (CE) loss, which is a generalized loss function appropriate for multinomial data. It is calculated as:

$$-\underset{i}{\sum }C\left(i\right)\log\;P\left(i\right)$$

where *C*(*i*) is the probability of the reported cause of death (*i.e*., class probability) for the *i*th individual and *P*(*i*) is the predicted probability for having that cause of death. It assesses the degree to which an outcome can be appropriately divided into distinct groups, with a lower number indicating a better performance. Unlike other clinical evaluation metrics, CE loss does not have a direct clinical interpretation. To understand the quality of the overall classification, we calculated the baseline CE loss as a comparison, which is based on a random guess model. In addition, we calculated the one versus rest area under the receiver operator characteristic curve (OvR AUC) for each of the outcomes. In OvR AUC, one calculates the area under the receiver operating characteristic curve for each outcome (*i.e*., cause of death) compared with all other possibilities.^19^ It has a similar interpretation as the traditional area under the receiver operating characteristic curve as a discrimination metric. Here, it assesses the degree to which we can distinguish each particular cause of death from the other causes of death. We report bootstrap-based 95% confidence intervals.

#### Identifying Risk Markers for Cause-Specific Death

We next assessed which clinical factors were important markers for each cause of death. For each cause of death, we used the appropriate case and control sample. We fit a Ridge logistic regression using the 53 covariates (see Supplemental Table 3) to identify important features. We choose a Ridge (instead of a LASSO) variable selection model so that variables would not be shrunk to 0 and the effect of all covariates could be compared. Results were similar in sensitivity analyses using a LASSO fitted model. To allow for the comparison of predictor variables, each was scaled to have unit variance before fitting the Ridge model. We compared model coefficients across each of the outcomes both visually and by assessing the correlation of the coefficient vectors. We also calculated the variability of each set of coefficients across causes of death. We note that models may include collinear predictor variables, and these coefficients do not have a causal interpretation. Instead, the coefficients simply represent a quantifiable measure of the relationship to the outcome and the variables may be most appropriately thought of as risk markers.

All analysis were conducted using R 4.0.2, we used the “XgBoost” and “glmnet” for the classification models.^18,20,21^ We computed SMD from the “tableone” package by computing the average of all pairwise SMDs.^22^ The 95% confidence interval for OvR AUCs were calculated from the pROC package.^23^

## Results

We identified 19,793 individuals who met the inclusion criteria of at least one encounter at a DCI clinic in the 30 days before they died. Most notably, patients with SCD were younger and patients with unknown cause of death were older (Table 1). Patients with a history of atherosclerotic cardiovascular disease were over-represented among those with SCD, non-SCD cardiovascular death, and unknown deaths, which includes dialysis withdrawal. Patients with a history of cancer were over-represented among other deaths, which includes death from malignancy. In sensitivity analyses using the NDI cause of death, there were 5666 individuals. Using NDI, patients with a history of atherosclerotic cardiovascular disease were also over-represented among those with SCD, non-SCD cardiovascular death, and unknown deaths (Supplemental Table 5). Multiple comorbidities were enriched among those with deaths classified as unknown, including cancer, liver disease, peripheral vascular disease, stroke, and gastrointestinal disease, although a lower percentage of deaths were classified in this group (9% with NDI versus 23% with USRDS).

**Table 1:** Patient characteristics by USRDS cause of death

Characteristic	Overall	SCD	Non-SCD Cardiovascular	Infection	Others	Unknown	SMD
*N*	19,793	6810	2761	1887	3704	4631	—
Age at death, median (IQR)	71 (61–80)	69 (60–78)	72 (62–79)	71 (61–79)	71 (61–79)	74 (65–82)	0.14
Male sex, *n* (%)	11,039 (55.8)	3857 (56.6)	1557 (56.4)	1012 (53.6)	2086 (56.3)	2527 (54.6)	0.03
**Race and ethnicity, *n* (%)**							0.15
Hispanic	912 (4.6)	330 (4.8)	145 (5.3)	84 (4.5)	142 (3.8)	211 (4.6)	—
Non-Hispanic Black	5788 (29.2)	2268 (33.3)	782 (28.3)	594 (31.5)	1125 (30.4)	1019 (22.0)	—
Non-Hispanic White	12,435 (62.8)	3981 (58.5)	1743 (63.1)	1117 (59.2)	2320 (62.6)	3274 (70.7)	—
Others	658 (3.3)	231 (3.4)	91 (3.3)	92 (4.9)	117 (3.2)	127 (2.7)	—
Time (yr) since ESKD incidence to outcome, median (IQR)	2.31 (1.00–4.27)	2.51 (1.14–4.52)	2.25 (1.01–4.14)	2.23 (0.92–4.38)	2.09 (0.91–4.02)	2.23 (0.95–4.14)	0.06
**USRDS claims available^a^**, ***n* (%)**	16,047 (81.1)	5498 (80.7)	2326 (84.2)	1519 (80.5)	2994 (80.8)	3710 (80.1)	0.05
Atherosclerotic heart disease	12,825 (79.9)	4480 (81.5)	1978 (85.0)	1184 (77.9)	2239 (74.8)	2944 (79.4)	0.12
Congestive heart failure	13,481 (84.0)	4641 (84.4)	2054 (88.3)	1270 (83.6)	2394 (80.0)	3122 (84.2)	0.10
CVA/TIA	9702 (60.5)	3261 (59.3)	1500 (64.5)	924 (60.8)	1718 (57.4)	2299 (62.0)	0.07
PVD	12,929 (80.6)	4461 (81.1)	1850 (79.5)	1299 (85.5)	2302 (76.9)	3017 (81.3)	0.10
Other cardiac	13,447 (83.8)	4612 (83.9)	1989 (85.5)	1311 (86.3)	2429 (81.1)	3106 (83.7)	0.07
COPD	9801 (61.1)	3331 (60.6)	1404 (60.4)	948 (62.4)	1811 (60.5)	2307 (62.2)	0.02
Gastrointestinal	6535 (40.7)	2204 (40.1)	885 (38.0)	658 (43.3)	1237 (41.3)	1551 (41.8)	0.05
Liver disease	4154 (25.9)	1372 (25.0)	540 (23.2)	457 (30.1)	829 (27.7)	956 (25.8)	0.08
Dysrhythmia	13,285 (82.8)	4698 (85.4)	1958 (84.2)	1287 (84.7)	2311 (77.2)	3031 (81.7)	0.10
Cancer	4517 (28.1)	1280 (23.3)	496 (21.3)	371 (24.4)	1314 (43.9)	1056 (28.5)	0.22
Diabetes	13,173 (82.1)	4610 (83.8)	1924 (82.7)	1285 (84.6)	2320 (77.5)	3034 (81.8)	0.08

COPD, chronic obstructive pulmonary disease; CVA/TIA, cerebrovascular accident/transient ischemic attack; IQR, interquartile range; PVD, peripheral vascular disease; SCD, sudden cardiac death; SMD, standardized mean difference; USRDS, United States Renal Data System.

a Following rows are *N* (%) of patients ever had comorbidity, among patients with USRDS claims.

### Classifying Cause of Death

**Figure 1:**
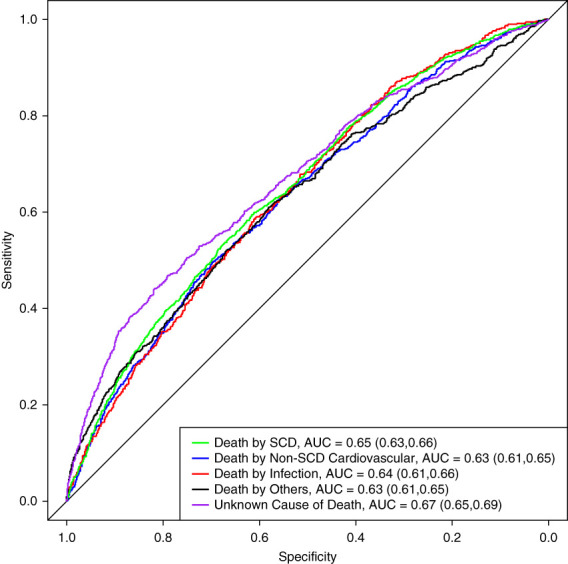
**OvR AUC for USRDS cause of death.** Receiver operating characteristic curves are displayed for classification of each cause of death versus all other using a XgBoost multiclass model. AUCs and their 95% confidence intervals are provided in legend. AUC, area under the receiver operating characteristic curve; OvR, one versus rest; SCD, sudden cardiac death; USRDS, United States Renal Data System; XgBoost, Extreme Gradient Boosting.

Based on the frequencies of the five causes of death groups, the baseline CE loss is 1.52. When fitting the XgBoost model this decreased to 1.44, consistent with a relatively small improvement. For the NDI based outcomes, the baseline was 1.45 with an improvement to 1.39. The OvR AUC are shown for the USRDS and NDI-based outcomes in Figure 1 and Supplemental Figure 1, respectively. All OvR AUC were between 0.59 and 0.70, suggesting minimal ability to distinguish the probability of one cause of death from another. Of note, the unknown cause of death had the qualitatively highest OvR AUC. To evaluate whether discrimination was higher for certain outcomes within subgroups of individuals, we performed age-stratified analysis to compare the classification separately between younger and older individuals with no difference in performance (Supplemental Table 6).

### Factors Related to Each Cause of Death

**Figure 2:**
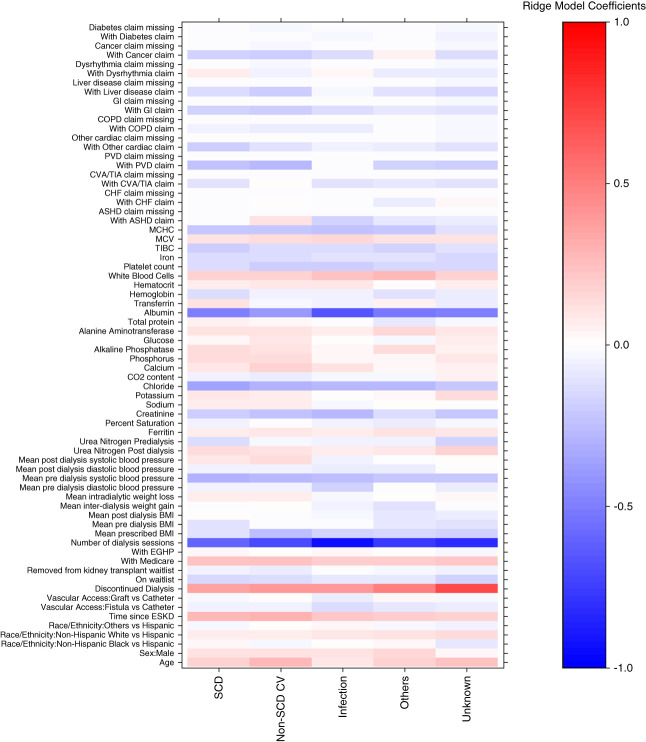
**Ridge coefficients for USRDS cause of death using nested case-control binary outcome models.** Coefficients between 0 and 1 (red) are directly related to the cause of death. Coefficients between 0 and −1 (blue) are inversely related to the cause of death. Coefficients are derived from a ridge logistic regression with standardized predictor variables and comparison of each case (death from labeled cause of death) compared with a control alive at the same time point. Coefficients are displayed across different cause of death models demonstrating similar direction and magnitude of coefficients across different models. ASHD, atherosclerotic heart disease; BMI, body mass index; CHF, congestive heart failure; COPD, chronic obstructive pulmonary disease; CV, cardiovascular; CVA/TIA, cerebrovascular accident/transient ischemic attack; EGHP, employer group health plan insurance; GI, gastrointestinal; MCHC, mean corpuscular hemoglobin concentration; MCV, mean corpuscular volume; PVD, peripheral vascular disease; TIBC, total iron binding capacity.

Supplemental Table 7 shows the characteristics of the control populations created for each cause of death. As expected, control participants characteristics are all fairly similar, with all SMDs <0.05. The ridge-based coefficients from the case-control models were relatively consistent across the different causes of death, suggesting that each cause of death has shared risk markers (Figure 2). The identified risk markers include many well-known clinical factors such as low serum albumin, low predialysis systolic BP, high white blood cell count, and dialysis vintage, among others. The risk markers with the largest variability across outcomes were discontinued status (*i.e*., withdrawal from hemodialysis), number of sessions in the previous month, claim for atherosclerotic heart disease, claim for peripheral vascular disease, and serum albumin level. The actual values for all coefficients are provided in Supplemental Table 8 for reference. Results were similar for the NDI-based outcome (Supplemental Figure 2 and Supplemental Table 9).

**Figure 3:**
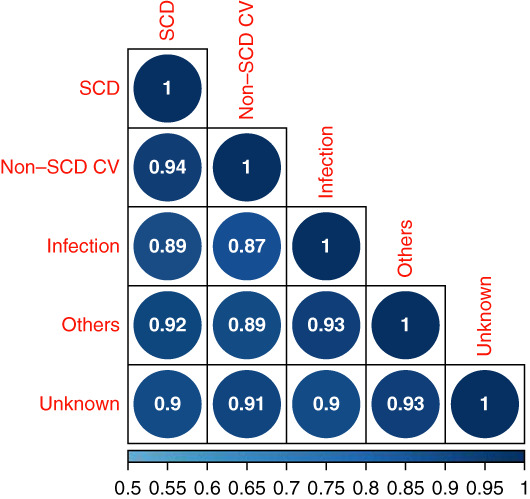
**Correlation plot for ridge coefficients for USRDS cause of death using nested case-control binary outcome models.** Coefficients are derived from a ridge logistic regression with standardized predictor variables and comparison of each case (death from labeled cause of death) compared with a control alive at the same time point. Correlation across all coefficients is displayed comparing each pair of models and demonstrating very high correlation across all pairings. CV, cardiovascular; SCD, sudden cardiac death.

Finally, to provide summarized quantitative assessment of the similarity of the case-control models for different causes of death, we compared the correlation of the full set of coefficient values for each covariate across models. As depicted in Figure 3, the coefficients were all highly correlated with values ranging from 0.87 to 0.94. The highest correlation was between non-SCD cardiovascular cause of death and SCD. Again, results were similar for the NDI outcome (Supplemental Figure 3).

## Discussion

In this study, we were unable to identify clinical factors that meaningfully distinguished among patients on maintenance hemodialysis who die of different causes within the next 30 days. Using an alternative case-control approach to model each cause separately, we found that the model parameters were quite similar even for different causes. Our group has recently reported poor agreement between USRDS and NDI sources of cause of death information for patients treated with maintenance hemodialysis.^16^ Our inability to detect distinct clinical risk phenotypes, interpreted along with this prior result, may suggest that the cause of death information currently available in USRDS and NDI is not precise. This would have important implications for the use of administratively reported cause of death as an outcome in interventional or observational studies in nephrology. Alternatively, our results may indicate a general risk phenotype for patients on maintenance hemodialysis that increases vulnerability to a wide array of final causes of death. This would also be an important conclusion because it may suggest that it would be difficult to individualize or target treatment strategies to certain phenotypic groups (*e.g*., implantable cardiac defibrillators for patients with an SCD risk phenotype). Another final possibility is that there are distinct risk phenotypes, but we lacked important clinical information, including novel biomarkers, that would be needed to distinguish among causes of death. Future research could test this final hypothesis with additional biomarker studies.

We hypothesized that certain clinical features would segregate with causes of death, providing a clinical risk phenotype that could be used for personalized dialysis care. For instance, we anticipated that use of central venous catheters and inflammatory markers, such as high white blood cell count might distinguish patients at risk for death from infection, while intradialytic hypotension and cardiovascular comorbidities might distinguish patients at risk for death from cardiovascular disease, and low body weight and nutritional indices might distinguish patients with other deaths that include malnutrition/cachexia. Our results may suggest that there is a common failure to thrive or frailty phenotype that precedes many types of deaths among maintenance hemodialysis patients. This is supported by extensive clinical studies demonstrating that body weight, serum albumin, and even paradoxical risk markers, such as low-normal BP, are among the top factors related to all-cause death in this population.^24^ Furthermore, interventional trials focused on specific pathophysiologic processes leading to CVD, such as hyperlipidemia and arrhythmia, have not been successful in hemodialysis.^25–27^ It may be more useful to consider most deaths among hemodialysis patients in a more complex manner. Rather than attributing deaths to distinct causes, it is likely that deaths are related to many synergizing factors in kidney failure, which include coexisting disease and the uremic milieu. A recent study by Stedman *et al.*^28^ demonstrated a striking increase in mortality among patients with kidney failure on dialysis with only 31% relative survival compared with similarly aged adults in the general population. Inclusion of comorbidities did not explain this risk, suggesting that these excess deaths may best be attributed to kidney failure as opposed to other causes. We acknowledge that the identification of intervenable risk factors was not the focus of this study and would require a different study design and approach. Although this is an important question, the coefficients of the models that we present cannot be used to infer whether certain variables may increase or decrease the risk of death from a specific cause. Instead, our study suggests that patients with kidney failure who die of different causes have similar clinical features in the month before death, best interpreted as risk markers.

Our findings also raise the concern that the cause of death indicated in USRDS (Form CMS 2746) and NDI may have limited face validity in patients on hemodialysis. Our team’s prior work has demonstrated high levels of discordance between the primary cause of death between these sources.^16^ In particular, SCD was much more commonly attributed in the USRDS than in the NDI, which could be a result of different expectations and biases among dialysis providers. In the few studies available with electrocardiographic monitoring, it has been demonstrated that bradycardia and asystolic pauses are the most common abnormal heart rhythms observed in prevalent hemodialysis patients, as opposed to ventricular and atrial tachyarrhythmias typically thought of as causes of SCD in the general population.^29,30^ Our inability to identify clinical features distinguishing causes of death in this study may underscore the uncertainty and disparate definitions used for the cause of death in most current reporting. Imprecise definitions may jeopardize research into risk factors for specific causes of death (*e.g*., SCD^11,31^) by leading to missed or underestimated effects because of misclassification.

Our study has many strengths, including a large population of patients with detailed EHR data and unique linkages to USRDS and NDI defined cause of death. We used a variety of approaches to interrogate clinical risk phenotypes. We also had several important limitations. We did not have gold-standard information on cause of death as obtained by adjudication or autopsy. Many patients may also have several causes of death, including underlying and immediate causes. The 77 unique causes of death were categorized into five distinct groups. There was large heterogeneity of causes in the group of other deaths, which could include diverse etiologies such as cancer, hemorrhage or liver disease. Our ability to identify risk phenotypes for these less common causes of death may have been limited by pooling within this heterogenous group. We used the primary cause of death as determined by health care providers. Furthermore, we are limited by the clinical variables that are available in dialysis EHR data. While existing data include many biometric and laboratory variables, it may not include many pathophysiologically informative biomarkers that are not routinely measured in dialysis care. These could include cardiac injury markers that have been effective in other studies (*e.g*., troponin, NT-proBNP), inflammatory markers (*e.g*., IL-6), imaging data, electrocardiograms, and echocardiography, among others.^32,33^ Furthermore, our focus on the 30 days before death may not have provided a sufficient window to assess changes in health status that could be informative.^34^ To ensure clinical information, we only included patients with a DCI encounter in the 30 days before death. We did not have in-hospital data; thus, our results may not be generalizable to the inpatient setting. Nonetheless, our findings suggest that we are unlikely to be able to differentiate clinical risk phenotypes using exclusively the data found within dialysis EHRs. Future studies should consider integrating dialysis EHRs with broader health records from a variety of inpatient and outpatient sources to develop a more complete picture of health status.

In summary, despite access to detailed dialysis data and use of state-of-the-art machine learning statistical methods, we were not able to distinguish among causes of death in maintenance hemodialysis patients using clinically available EHR variables.

## Supplementary Material

SUPPLEMENTARY MATERIAL

## Data Availability

Partial restrictions to the data and/or materials apply. Data for this study were provided by the USRDS, NDI, and DCI. EHR data from DCI and linked data files cannot be directly shared by the study team based on the terms of data use agreements. USRDS files that were used for this analysis can be requested from the USRDS at: https://www.niddk.nih.gov/about-niddk/strategic-plans-reports/usrds.

## Supplemental Material

Supplemental Table 1. USRDS causes of death definitions.

Supplemental Table 2. NDI causes of death definitions.

Supplemental Table 3. Variable definitions.

Supplemental Table 4. Degree of missingness for predictor variables.

Supplemental Table 5. Patient characteristics by NDI cause of death.

Supplemental Table 6. Comparison of classification in younger and older adults.

Supplemental Table 7. Patient characteristics for control patient-months.

Supplemental Table 8. Ridge coefficients for USRDS.

Supplemental Table 9. Ridge coefficients for NDI.

Supplemental Figure 1. One versus rest area under the receiver operating curve (AUC) for National Death Index (NDI) cause of death.

Supplemental Figure 2. Ridge coefficients for National Death Index (NDI) cause of death using nested case-control binary outcome models.

Supplemental Figure 3. Correlation plot for ridge coefficients for National Death Index (NDI) cause of death using nested case-control binary outcome models.
