# Supplementary material for: Distinguishing Among Causes of Death for Patients with Kidney Failure on Hemodialysis
Source: Kidney360. 2024 Dec 16;6(3):432–40. doi: 10.34067/KID.0000000681 (PMC11970861; doi:10.34067/KID.0000000681)
Supplement: SUPPLEMENTARY MATERIAL [file kidney360-6-432-s001.pdf]

ASN American Society of Nephrology · JASN Journal of the American Society of Nephrology · KIDNEY360 Accessing Our World From Every Angle · CJASN Clinical Journal of the American Society of Nephrology

ASN Journal Disclosure Form

As per ASN journal policy, I have disclosed any financial relationships or commitments I have held in the past 36 months as included below. I have listed my Current Employer below to indicate there is a relationship requiring disclosure. If no relationship exists, my Current Employer is not listed.

P. Ephraim reports the following:
Employer: Feinstein Institute for Medical Research, Northwell Health; and Consultancy: Stony Run Consulting.

I understand that the information above will be published within the journal article, if accepted, and that failure to comply and/or to accurately and completely report the potential financial conflicts of interest could lead to the following: 1) Prior to publication, article rejection, or 2) Post-publication, sanctions ranging from, but not limited to, issuing a correction, reporting the inaccurate information to the authors' institution, banning authors from submitting work to ASN journals for varying lengths of time, and/or retraction of the published work.

Name: Patti Ephraim
Manuscript ID: K360-2024-000518R1
Manuscript Title: Distinguishing Among Causes of Death for Patients with Kidney Failure on Hemodialysis
Date of Completion: October 22, 2024
Disclosure Updated Date: May 22, 2024

ASN American Society of Nephrology
JASN Journal of the American Society of Nephrology
KIDNEY360 Accessing Our World From Every Angle
CJASN Clinical Journal of the American Society of Nephrology

ASN Journal Disclosure Form

As per ASN journal policy, I have disclosed any financial relationships or commitments I have held in the past 36 months as included below. I have listed my Current Employer below to indicate there is a relationship requiring disclosure. If no relationship exists, my Current Employer is not listed.

B. Goldstein reports the following:
Employer: Duke Univeristy

I understand that the information above will be published within the journal article, if accepted, and that failure to comply and/or to accurately and completely report the potential financial conflicts of interest could lead to the following: 1) Prior to publication, article rejection, or 2) Post-publication, sanctions ranging from, but not limited to, issuing a correction, reporting the inaccurate information to the authors' institution, banning authors from submitting work to ASN journals for varying lengths of time, and/or retraction of the published work.

Name: Benjamin A. Goldstein
Manuscript ID: K360-2024-000518R1
Manuscript Title: Distinguishing Among Causes of Death for Patients with Kidney Failure on Hemodialysis
Date of Completion: November 14, 2024
Disclosure Updated Date: November 14, 2024

ASN American Society of Nephrology
JASN Journal of the American Society of Nephrology
KIDNEY360 Accessing Our World From Every Angle
CJASN Clinical Journal of the American Society of Nephrology

ASN Journal Disclosure Form

As per ASN journal policy, I have disclosed any financial relationships or commitments I have held in the past 36 months as included below. I have listed my Current Employer below to indicate there is a relationship requiring disclosure. If no relationship exists, my Current Employer is not listed.

J. Scialla reports the following:
Employer: University of Virginia; and Advisory or Leadership Role: Deputy Editor, American Journal of Kidney Diseases (paid); Scientific Advisory Board, National Kidney Foundation (unpaid).

I understand that the information above will be published within the journal article, if accepted, and that failure to comply and/or to accurately and completely report the potential financial conflicts of interest could lead to the following: 1) Prior to publication, article rejection, or 2) Post-publication, sanctions ranging from, but not limited to, issuing a correction, reporting the inaccurate information to the authors' institution, banning authors from submitting work to ASN journals for varying lengths of time, and/or retraction of the published work.

Name: Julia J. Scialla
Manuscript ID: K360-2024-000518R1
Manuscript Title: Distinguishing Among Causes of Death for Patients with Kidney Failure on Hemodialysis
Date of Completion: October 21, 2024
Disclosure Updated Date: April 30, 2024

ASN Journal Disclosure Form

As per ASN journal policy, I have disclosed any financial relationships or commitments I have held in the past 36 months as included below. I have listed my Current Employer below to indicate there is a relationship requiring disclosure. If no relationship exists, my Current Employer is not listed.

T. Shafi reports the following:
Consultancy: Allucent (DSMB Member); and Research Funding: Inactive: Clinical site investigator (Numares).

I understand that the information above will be published within the journal article, if accepted, and that failure to comply and/or to accurately and completely report the potential financial conflicts of interest could lead to the following: 1) Prior to publication, article rejection, or 2) Post-publication, sanctions ranging from, but not limited to, issuing a correction, reporting the inaccurate information to the authors' institution, banning authors from submitting work to ASN journals for varying lengths of time, and/or retraction of the published work.

Name: Tariq Shafi
Manuscript ID: K360-2024-000518R1
Manuscript Title: Distinguishing Among Causes of Death for Patients with Kidney Failure on Hemodialysis
Date of Completion: October 22, 2024
Disclosure Updated Date: October 21, 2024

ASN
American Society of Nephrology
JASN
Journal of the American Society of Nephrology
KIDNEY360
Accessing Our World From Every Angle
CJASN
Clinical Journal of the American Society of Nephrology

ASN Journal Disclosure Form

As per ASN journal policy, I have disclosed any financial relationships or commitments I have held in the past 36 months as included below. I have listed my Current Employer below to indicate there is a relationship requiring disclosure. If no relationship exists, my Current Employer is not listed.

M. Tran reports the following:
Employer: University of Virginia

I understand that the information above will be published within the journal article, if accepted, and that failure to comply and/or to accurately and completely report the potential financial conflicts of interest could lead to the following: 1) Prior to publication, article rejection, or 2) Post-publication, sanctions ranging from, but not limited to, issuing a correction, reporting the inaccurate information to the authors' institution, banning authors from submitting work to ASN journals for varying lengths of time, and/or retraction of the published work.

Name: Michelle Tran
Manuscript ID: K360-2024-000518R1
Manuscript Title: Distinguishing Among Causes of Death for Patients with Kidney Failure on Hemodialysis
Date of Completion: November 14, 2024
Disclosure Updated Date: November 14, 2024

ASN American Society of Nephrology | JASN Journal of the American Society of Nephrology | KIDNEY360 Accessing Our World From Every Angle | CJASN Clinical Journal of the American Society of Nephrology

ASN Journal Disclosure Form

As per ASN journal policy, I have disclosed any financial relationships or commitments I have held in the past 36 months as included below. I have listed my Current Employer below to indicate there is a relationship requiring disclosure. If no relationship exists, my Current Employer is not listed.

D. Weiner reports the following:
Employer: Tufts Medical Center Physicians Organization; Research Funding: All compensation paid to Tufts MC: Vertex (site PI); Cara (site PI, completed); CSL Behring (site PI, pending); Advisory or Leadership Role: Co Editor-in-Chief, NKF Primer on Kidney Diseases, 8th Edition; Medical Director of Clinical Research, Dialysis Clinic Inc; Councillor-at-Large, American Society of Nephrology; Member, Scientific Advisory Board, National Kidney Foundation; and Other Interests or Relationships: Member, Safety and Clinical Events Committee for "A Prospective, Multi-Center, Open-Label Assessment of Efficacy and Safety of Quanta SC+ for Home Hemodialysis" Trial (Avania CRO); Member, Adjudications Committee, ProKidney REACT Trial (WCG Clinical CRO).

I understand that the information above will be published within the journal article, if accepted, and that failure to comply and/or to accurately and completely report the potential financial conflicts of interest could lead to the following: 1) Prior to publication, article rejection, or 2) Post-publication, sanctions ranging from, but not limited to, issuing a correction, reporting the inaccurate information to the authors' institution, banning authors from submitting work to ASN journals for varying lengths of time, and/or retraction of the published work.

Name: Daniel E. Weiner
Manuscript ID: K360-2024-000518R1
Manuscript Title: Distinguishing Among Causes of Death for Patients with Kidney Failure on Hemodialysis
Date of Completion: October 22, 2024
Disclosure Updated Date: August 28, 2024

ASN American Society of Nephrology | JASN Journal of the American Society of Nephrology | KIDNEY360 Accessing Our World From Every Angle | CJASN Clinical Journal of the American Society of Nephrology

ASN Journal Disclosure Form

As per ASN journal policy, I have disclosed any financial relationships or commitments I have held in the past 36 months as included below. I have listed my Current Employer below to indicate there is a relationship requiring disclosure. If no relationship exists, my Current Employer is not listed.

J. Wilson reports the following:
Employer: Duke University

I understand that the information above will be published within the journal article, if accepted, and that failure to comply and/or to accurately and completely report the potential financial conflicts of interest could lead to the following: 1) Prior to publication, article rejection, or 2) Post-publication, sanctions ranging from, but not limited to, issuing a correction, reporting the inaccurate information to the authors' institution, banning authors from submitting work to ASN journals for varying lengths of time, and/or retraction of the published work.

Name: Jonathan A. Wilson
Manuscript ID: K360-2024-000518R1
Manuscript Title: Distinguishing Among Causes of Death for Patients with Kidney Failure on Hemodialysis
Date of Completion: October 22, 2024
Disclosure Updated Date: August 30, 2024

ASN American Society of Nephrology
JASN Journal of the American Society of Nephrology
KIDNEY360 Accessing Our World From Every Angle
CJASN Clinical Journal of the American Society of Nephrology

ASN Journal Disclosure Form

As per ASN journal policy, I have disclosed any financial relationships or commitments I have held in the past 36 months as included below. I have listed my Current Employer below to indicate there is a relationship requiring disclosure. If no relationship exists, my Current Employer is not listed.

C. Xu has nothing to disclose.

I understand that the information above will be published within the journal article, if accepted, and that failure to comply and/or to accurately and completely report the potential financial conflicts of interest could lead to the following: 1) Prior to publication, article rejection, or 2) Post-publication, sanctions ranging from, but not limited to, issuing a correction, reporting the inaccurate information to the authors' institution, banning authors from submitting work to ASN journals for varying lengths of time, and/or retraction of the published work.

Name: Chun Xu
Manuscript ID: K360-2024-000518R1
Manuscript Title: Distinguishing Among Causes of Death for Patients with Kidney Failure on Hemodialysis
Date of Completion: October 24, 2024
Disclosure Updated Date: May 22, 2024
