# Supplementary material for: Distinguishing Among Causes of Death for Patients with Kidney Failure on Hemodialysis
Source: Kidney360. 2024 Dec 16;6(3):432–40. doi: 10.34067/KID.0000000681 (PMC11970861; doi:10.34067/KID.0000000681)

Supplementary Materials for:

Distinguishing Among Causes of Death for Patients with Kidney Failure on Hemodialysis

Michelle Tran, MD[1], Chun Anna Xu, MB[2], Jonathan Wilson, MS[2], Patti L. Ephraim, MPH[3], Tariq Shafi, MBBS, MHS[4], Daniel E Weiner, MD[5], Benjamin A. Goldstein*, PhD[2] and Julia J. Scialla*, MD, MHS[1,6], on behalf of the Comparative Effectiveness Studies in Dialysis Patients Group

*denotes co-senior author

[1]Department of Medicine, University of Virginia School of Medicine, Charlottesville, VA [2]Department of Biostatistics and Bioinformatics, Duke University School of Medicine, Durham, NC; [3]Feinstein Institute for Medical Research, Northwell Health, New York, NY; [4]Department of Medicine, Division of Nephrology, Houston Methodist Hospital, Houston, TX; [5]Department of Medicine, Tufts Medical Center, Boston, MA, [6]Department of Public Health Sciences, University of Virginia School of Medicine, Charlottesville, VA;

Supplemental Table 1 – USRDS Causes of Death Definitions

Assigned category in this study	CMS Form 2746	USRDS definition
Sudden cardiac death (SCD)	28: Cardiac Arrhythmia	Arrhythmia/cardiac arrest
	29: Cardiac Arrest, Cause Unknown	Arrhythmia/cardiac arrest
Non-SCD cardiovascular death	23: Myocardial Infarction, Acute	AMI and ASHD
	25: Pericarditis, Incl. Cardiac Tamponade	Other cardiac
	26: Atherosclerotic Heart Disease	AMI and ASHD
	27: Cardiomyopathy	CHF
	30: Valvular Heart Disease	Other cardiac
	31: Pulmonary Edema Due to Exogenous Fluid	CHF
	32: Congestive heart failure	CHF
	36: Cerebro-Vascular Accident Including Intracranial Hemorrhage	CVA
	37: Ischemic Brain Damage/Anoxic Encephalopathy	CVA
Infectious death	33: Septicemia due to internal vascular access	Sepsis
	34: Septicemia due to vascular access catheter	Sepsis
	45: Peritoneal access infectious complication, bacterial	Other infection
	46: Peritoneal access infectious complication, fungal	Other infection
	47: Peritonitis (complication of peritoneal dialysis)	Other infection
	48: Central nervous system infection (brain abscess, meningitis, encephalitis, etc.)	Other infection
	49: Septicemia, Due to Vascular Access *Discontinued, as of Oct 2004	Other infection
	50: Septicemia, Due to Peritonitis	Sepsis
	51: Septicemia, Due to Peripheral Vascular Disease, Gangrene	Sepsis
	52: Septicemia, Other	Sepsis
	53: Pulmonary Infection (Bacterial)	Other infection
	54: Pulmonary Infection (Fungal))	Other infection
	55: Pulmonary Infection (Other)	Other infection
	56: Viral Infection, Cmv	Other infection
	57: Viral Infection, Other (Not 64 Or 65)	Other infection
	58: Tuberculosis	Other infection
	60: Infection, Other	Other infection

	61: Cardiac infection (endocarditis)	Other infection
	62: Pulmonary infection (pneumonia, influenza)	Other infection
	63: Abdominal infection (peritonitis-not complication of PD, perforated bowel, diverticular disease, gallbladder)	Other infection
	64: Hepatitis B	Other infection
	65: Other Viral Hepatitis	Other infection
	70: Genito-urinary infection (urinary tract infection, pyelonephritis, renal abscess)	Other infection
	71: Hepatitis C	Other infection
Other causes of death	82: Malignant Disease, Patient Ever On Immunosuppressive Therapy	Malignancy
	83: Malignant Disease (Not 82)	Malignancy
	24: Hyperkalemia	Hyperkalemia
	35: Pulmonary Embolus	Other
	38: Hemorrhage From Transplant Site	Other
	39: Hemorrhage From Vascular Access	Other
	41: Hemorrhage From Ruptured Vascular Aneurysm	Other
	42: Hemorrhage From Surgery (Not 38,39 Or 40)	Other
	43: Other Hemorrhage (Not Codes 38-42,72)	Other
	44: Mesenteric Infarction/Ischemic Bowel	Other
	66: Liver-Drug Toxicity	Other
	67: Cirrhosis	Other
	68: Polycystic Liver Disease	Other
	69: Liver Failure, Cause Unknown Other	Other
	72: Gastro-Intestinal Hemorrhage	Other
	73: Pancreatitis	Other
	75: Perforation Of Peptic Ulcer	Other
	76: Perforation Of Bowel (Not 75)	Other
	77: Hypokalemia	Other
	78: Hypernatremia	Other
	79: Hyponatremia	Other
	80: Bone Marrow Depression	Other
	81: Cachexia	Other
	84: Dementia, Incl. Dialysis Dementia, Alzheimer's	Other
	85: Seizures	Other

	87: Chronic Obstructive Lung Disease (COPD)	Other
	88: Complications Of Surgery	Other
	89: Air Embolism	Other
	90: Accident Related to Treatment	Other
	91: Accident Unrelated to Treatment	Other
	92: Suicide	Other
	93: Drug Overdose (Street Drugs)	Other
	94: Drug Overdose (Not 92 Or 93)	Other
	95: Acidosis	Other
	96: Adrenal insufficiency	Other
	97: Hypothyroidism	Other
	98: Other identified cause of death	Other
	100: Hypoglycemia	Other
	101: Hyperglycemia	Other
	102: Diabetic coma	Other
Unknown	99: Unknown	Unknown
	104: Withdrawal from dialysis/uremia	Withdrawal
	Missing or out of range of Death Notification form	Missing

Supplemental Table 2 – NDI Causes of Death Definitions

Assigned category in this study	ICD-10 code
Sudden cardiac death (SCD)	I46, I47.2, I49.0*, I49.3, I49.8, I49.9
Non-SCD cardiovascular death	I05-I11, I15, I20-I25, I34-I37, I42-I45, I47.0, I47.1, I47.9, I48, I49.1, I49.2, I49.4*, I49.5, I50, I51, I60-I74, I77-I79, I98-99, R02, K55
Infectious death	A00-B99, E06.0, G00-G08, I00-I01, I33, I38, J00-J06, J09-J18, J20-22, J36, J39.0, J39.1, J44.0, J85-J86, K35, K63.0, K65.0, K65.9, K80.0, K80.3, K80.4, K81, K83.0, L00-L08, M00-M02, M86, N30.0, N30.8, N41.0, N41.2, N41.3, O03.0, O03.5, O04.5, O07.0, O08.0, O75.3, O85-O86, R57.2, T80.2, T81.4, T82.6-T82.7, T83.5-T83.6, T84.5-T84.7, T85.7, T87.4, T88.0
Other causes of death	All other ICD-10 codes
Unknown	Missing ICD-10 code

Supplemental Table 3. Variable definitions

Variables	Type	Definition	Plausible Values
Age	Demographic	Patient's age at death	>18
Sex	Demographic	Patient's sex	Male or Female
Waitlist status	Demographic	If patient on Kidney-Pancreas/Kidney transplant waitlist	Never On, On, Removed
Medicare	Demographic	If patient have Medicare	Yes/No
Employee Group Health Plan	Demographic	If patient have Employee Group Health Plan	Yes/No
Treatment Frequency	Encounter	The number of HD encounters patient have in 30 days before death	N/A
Vascular Access	Encounter	Vascular Access for hemodialysis	Fistula, graft, catheter
Duration of prior dialysis	Encounter	Time from patients' ESRD to end of service month	N/A
Prescribed BMI	Vital signs	Mean in the 30 days before death for prescribed HD BMI	N/A
Pre BMI	Vital signs	Mean in the 30 days before death for pre dialysis BMI	30 - 200 kg
Post BMI	Vital signs	Mean in the 30 days before death for post dialysis BMI	30 - 200 kg
Inter-dialytic weight gain	Vital signs	Mean weight gain from end of last dialysis	-10 - 10 kg
Intradialytic weight loss	Vital signs	Mean weight loss after dialysis session	-2 - 8 kg
Pre-dialysis blood pressure sitting diastolic	Vital signs	Mean pre dialysis diastolic blood pressure in the 30 days before death	30 - 150 mmHg
Pre-dialysis blood pressure sitting systolic	Vital signs	Mean pre dialysis systolic blood pressure in the 30 days before death	60 - 250 mmHg
Post-dialysis blood pressure sitting diastolic	Vital signs	Mean post dialysis diastolic blood pressure in the 30 days before death	30 - 150 mmHg
Post-dialysis blood pressure sitting systolic	Vital signs	Mean post dialysis systolic blood pressure in the 30 days before death	60 - 250 mmHg
Serum Urea Nitrogen Pre dialysis	Laboratory	Last serum urea nitrogen pre dialysis within 180 days before death	5 - 200 mg/dl
Serum Urea Nitrogen Post dialysis	Laboratory	Last serum urea nitrogen post dialysis within 180 days before death	5 - 200 mg/dl
Serum Ferritin	Laboratory	Last serum Ferritin within 180 days before death	1 - 4000 ng/ml
Serum Creatinine	Laboratory	Last Creatinine within 180 days before death	0 - 25 mg/dl
Serum Sodium	Laboratory	Last serum Sodium within 180 days before death	115 - 160 mEq/l
Serum Potassium	Laboratory	Last serum Potassium within 180 days before death	2 - 10 mEq/l
Serum Chloride	Laboratory	Last serum Chloride within 180 days before death	40 - 154 mEq/l
Serum CO2 Content	Laboratory	Last serum CO2 Content within 180 days before death	5 - 53 mEq/l
Serum Calcium	Laboratory	Last serum Calcium within 180 days before death	5 - 20 mg/dl
Serum Phosphorus	Laboratory	Last serum Phosphorus within 180 days before death	1 - 20 mg/dl
Serum Alkaline Phosphatase	Laboratory	Last serum ALK Phosphatase within 180 days before death	0 - 5280 IU/l
Serum Glucose	Laboratory	Last serum Glucose within 180 days before death	58 - 800 mg/dl

Serum Alanine Aminotransferase	Laboratory	Last ALT within 180 days before death	1 - 4330 U/l
Serum Total Protein	Laboratory	Last serum Total Protein within 180 days before death	1.7 - 14 mg/dl
Serum Albumin	Laboratory	Last serum Albumin BCG within 180 days before death	0 - 5.5 g/dl
Serum Transferrin	Laboratory	Last Transferrin within 180 days before death	93 - 279 mg/dl
Hemoglobin	Laboratory	Last Hemoglobin within 180 days before death	3 - 20 g/dl
Hematocrit	Laboratory	Last Hematocrit within 180 days before death	6 - 60 %
Total White Blood Cells	Laboratory	Last White Blood Cells within 180 days before death	0 - 150 (x10^3/mcl)
Platelet Count	Laboratory	Last Platelet Count within 180 days before death	1 - 2000 (x10^3/mcl)
Serum Iron	Laboratory	Last Serum Iron within 180 days before death	20 - 178 mcg/dl
MCV	Laboratory	Last MCV within 180 days before death	70 - 145 fl
MCHC	Laboratory	Last MCHC within 180 days before death	12 - 45 mg/dl
Percent Saturation	Laboratory	Last Percent Saturation within 180 days before death	0 - 100 %
Serum TIBC	Laboratory	Last serum TIBC within 180 days before death	129 - 850 mcg/dl
Ever Arteriosclerotic heart disease	Claims	If patient ever had ASHD claim	Yes; No; N/A*
Ever Congestive heart failure	Claims	If patient ever had CHF claim	Yes; No; N/A*
Ever cerebral vascular accident/Transient Ischemic Attack	Claims	If patient ever had CVA/TIA claim	Yes; No; N/A*
Ever Peripheral vascular disease	Claims	If patient ever had PVD claim	Yes; No; N/A*
Ever Other Cardiac disease	Claims	If patient ever had other cardiac disease claim	Yes; No; N/A*
Ever Chronic obstructive pulmonary disease	Claims	If patient ever had COPD claim	Yes; No; N/A*
Ever GI	Claims	If patient ever had GI claim	Yes; No; N/A*
Ever Liver Disease	Claims	If patient ever had liver disease claim	Yes; No; N/A*
Ever Dysrhythmia	Claims	If patient ever had dysrhythmia claim	Yes; No; N/A*
Ever Cancer	Claims	If patient ever had cancer claim	Yes; No; N/A*
Ever Diabetes	Claims	If patient ever had diabetes claim	Yes; No; N/A*

* N/A means Medicare claims were not available at time of death

Supplemental Table 4. Degree of Missingness for Predictor Variables

Variables	Percent of missing before LOCF, USRDS	Percent of missing before LOCF, NDI
Age	0	0
Sex	0	0
Waitlist status	0	0
Medicare	0	0
Employee Group Health Plan	0	0
Treatment Frequency	0	0
Vascular Access	0	0
Duration of prior dialysis	0	0
Prescribed BMI	6.40	5.61
Pre BMI	3.41	2.89
Post BMI	3.63	3.16
Inter-dialytic weight gain	1.05	1.09
Intradialytic weight loss	0.39	0.58
Pre-dialysis blood pressure sitting diastolic	0.63	0.19
Pre-dialysis blood pressure sitting systolic	0.61	0.19
Post-dialysis blood pressure sitting diastolic	0.72	0.26
Post-dialysis blood pressure sitting systolic	0.70	0.25
Serum Urea Nitrogen Pre dialysis	1.43	1.80
Serum Urea Nitrogen Post dialysis	5.32	6.18
Serum Ferritin	3.72	3.94
Serum Creatinine	1.63	2.31
Serum Sodium	1.68	2.45
Serum Potassium	1.50	2.01
Serum Chloride	1.66	2.44
Serum CO2 Content	1.64	2.33
Serum Calcium	1.51	1.92
Serum Phosphorus	1.65	2.19
Serum Alkaline Phosphatase	1.64	2.35
Serum Glucose	2.97	4.06
Serum Alanine Aminotransferase	2.09	3.48
Serum Total Protein	1.69	2.52
Serum Albumin	1.48	1.94
Serum Transferrin	16.30	18.51
Hemoglobin	1.54	1.82
Hematocrit	1.49	1.87
Total White Blood Cells	1.98	2.31
Platelet Count	1.79	2.84
Serum Iron	8.02	12.48
MCV	1.80	2.74
MCHC	1.84	2.91
Percent Saturation	22.25	71.06
Serum TIBC	28.62	72.48
Ever Arteriosclerotic heart disease	18.93	15.30
Ever Congestive heart failure	18.93	15.30
Ever cerebral vascular accident/Transient Ischemic Attack	18.93	15.30
Ever Peripheral vascular disease	18.93	15.30
Ever Other Cardiac disease	18.93	15.30
Ever Chronic obstructive pulmonary	18.93	15.30

disease		
Ever GI	18.93	15.30
Ever Liver Disease	18.93	15.30
Ever Dysrhythmia	18.93	15.30
Ever Cancer	18.93	15.30
Ever Diabetes	18.93	15.30

Supplemental Table 5. Patient Characteristics by NDI cause of death

	Overall	SCD	Non-SCD Cardiovascular	Infection	Others	Unknown	SMD
N (%) or median [IQR]	5,666	1,037	1,164	562	2,372	531	
Age at death (median [IQR])	72 [61, 80]	70 [59, 79]	71 [62, 79]	71 [61, 79]	73 [63, 81]	69 [59, 78]	0.12
Male Sex	3,096 (54.6%)	575 (55.4%)	667 (57.3%)	306 (54.4%)	1,247 (52.6%)	301 (56.7%)	0.05
Race/Ethnicity							0.11
Hispanic	212 (3.7%)	28 (2.7%)	41 (3.5%)	17 (3.0%)	98 (4.1%)	28 (5.3%)	
Non-Hispanic Black	1,531 (27.0%)	314 (30.3%)	312 (26.8%)	168 (29.9%)	580 (24.5%)	157 (29.6%)	
Non-Hispanic White	3,783 (66.8%)	664 (64.0%)	786 (67.5%)	363 (64.6%)	1,632 (68.8%)	338 (63.7%)	
Years to outcome (median[IQR])	1.3 [0.6, 2.4]	1.4 [0.6, 2.3]	1.3 [0.6, 2.4]	1.1 [0.5, 2.2]	1.2 [0.6, 2.3]	2.2 [1.1, 3.5]	0.28
USRDS claims available (% available) *	4799 (84.7%)	893 (86.1%)	990 (85.1%)	464 (82.6%)	1995 (84.1%)	457 (86.1%)	0.05
ASHD	3,541 (73.8%)	666 (74.6%)	796 (80.4%)	315 (67.9%)	1,394 (69.9%)	370 (81.0%)	0.17
CHF	3,849 (80.2%)	711 (79.6%)	836 (84.4%)	371 (80.0%)	1,542 (77.3%)	389 (85.1%)	0.11
CVATIA	2,681 (55.9%)	456 (51.1%)	590 (59.6%)	244 (52.6%)	1,092 (54.7%)	299 (65.4%)	0.15
PVD	3,484 (72.6%)	642 (71.9%)	728 (73.5%)	340 (73.3%)	1,397 (70.0%)	377 (82.5%)	0.13
Other Cardiac	3,752 (78.2%)	677 (75.8%)	800 (80.8%)	373 (80.4%)	1,517 (76.0%)	385 (84.2%)	0.11
COPD	2,616 (54.5%)	459 (51.4%)	537 (54.2%)	255 (55.0%)	1,094 (54.8%)	271 (59.3%)	0.07
GI	1,668 (34.8%)	290 (32.5%)	320 (32.3%)	183 (39.4%)	680 (34.1%)	195 (42.7%)	0.12
Liver Disease	933 (19.4%)	155 (17.4%)	152 (15.4%)	111 (23.9%)	373 (18.7%)	142 (31.1%)	0.18
Dysrhythmia	3,696 (77.0%)	715 (80.1%)	784 (79.2%)	364 (78.4%)	1,454 (72.9%)	379 (82.9%)	0.11
Cancer	1,224 (25.5%)	193 (21.6%)	186 (18.8%)	89 (19.2%)	635 (31.8%)	121 (26.5%)	0.16
Diabetes	3,720 (77.5%)	708 (79.3%)	774 (78.2%)	370 (79.7%)	1,497 (75.0%)	371 (81.2%)	0.07
USRDS Cause of Death, N(%)							0.65
Unknown	1,272 (22.4%)	164 (15.8%)	203 (17.4%)	99 (17.6%)	679 (28.6%)	127 (23.9%)	
Non-SCD Cardiovascular	923 (16.3%)	152 (14.7%)	395 (33.9%)	60 (10.7%)	236 (9.9%)	80 (15.1%)	
Infection	568 (10.0%)	72 (6.9%)	57 (4.9%)	205 (36.5%)	161 (6.8%)	73 (13.7%)	
SCD	1,818 (32.1%)	522 (50.3%)	386 (33.2%)	113 (20.1%)	631 (26.6%)	166 (31.3%)	
Others	1,085 (19.1%)	127 (12.2%)	123 (10.6%)	85 (15.1%)	665 (28.0%)	85 (16.0%)	

*: Following rows are N(%) of patients ever had comorbidity, among patients with USRDS claims
SCD, sudden cardiac death; SMD, standardized mean difference; IQR, interquartile range; CVA/TIA, cerebrovascular accident/transient ischemic attack; PVD, peripheral vascular disease; COPD, chronic obstructive pulmonary disease; GI, gastrointestinal

Supplemental Table 6. Comparison of classification in younger and older adults

**Sensitivity Analysis using USRDS labels**		
	AUC- model with patients <65 years old	AUC- model with patients >=65 years old
SCD	0.64 (0.61, 0.67)	0.63 (0.61, 0.65)
Cardiovascular	0.58 (0.55, 0.62)	0.62 (0.60, 0.65)
Infection	0.65 (0.60, 0.69)	0.61 (0.58, 0.64)
Others	0.63 (0.59, 0.67)	0.61 (0.59, 0.64)
Unknown	0.62 (0.58, 0.65)	0.66 (0.64, 0.68)
**Sensitivity Analysis using NDI labels**		
	AUC- model with patients <65 years old	AUC- model with patients >=65 years old
SCD	0.55 (0.49, 0.62)	0.57 (0.52, 0.62)
Cardiovascular	0.59 (0.52, 0.65)	0.61 (0.57, 0.66)
Infection	0.68 (0.60, 0.76)	0.58 (0.52, 0.63)
Others	0.57 (0.51, 0.62)	0.62 (0.59, 0.65)
Unknown	0.71 (0.63, 0.79)	0.69 (0.63, 0.74)

Supplemental Table 7. Patient Characteristics for Control patient-months

	Overall Control	Control for SCD	Control for Non-SCD Cardiovascular	Control for Infection	Control for Others	Control for Unknown	SMD
N (%) or median [IQR]	19,793	6,810	2,761	1,887	3,704	4,631	
Age at death (median [IQR])	64 [53, 73]	64 [53, 74]	64 [53, 74]	64 [53, 74]	63 [53, 73]	63 [53, 73]	0.02
Male Sex (%)	10,841 (54.8%)	3,749 (55.1%)	1,478 (53.5%)	1,045 (55.4%)	1,973 (53.3%)	2,596 (56.1%)	0.03
Race/Ethnicity (%)							0.04
Hispanic	1,378 (7.0%)	487 (7.2%)	195 (7.1%)	121 (6.4%)	241 (6.5%)	334 (7.2%)	
Non-Hispanic Black	8,237 (41.6%)	2,804 (41.2%)	1,115 (40.4%)	811 (43.0%)	1,563 (42.2%)	1,944 (42.0%)	
Non-Hispanic White	9,219 (46.6%)	3,194 (46.9%)	1,332 (48.2%)	864 (45.8%)	1,712 (46.2%)	2,117 (45.7%)	
Years since ESRD incidence (median[IQR])	2.1 [0.9, 4.1]	2.2 [0.9, 4.1]	2.0 [0.8, 3.8]	2.2 [0.9, 4.2]	2.1 [0.9, 3.9]	2.2 [0.9, 4.2]	0.05
USRDS claims available (% available) *	17,056 (86.2%)	5,885 (86.4%)	2,379 (86.2%)	1,629 (86.3%)	3,170 (85.6%)	3,993 (86.2%)	0.01
ASHD	13,849 (81.2%)	4,822 (81.9%)	1,887 (79.3%)	1,331 (81.7%)	2,566 (80.9%)	3,243 (81.2%)	0.03
CHF	14,230 (83.4%)	4,933 (83.8%)	1,990 (83.6%)	1,356 (83.2%)	2,640 (83.3%)	3,311 (82.9%)	0.01
CVATIA	10,841 (63.6%)	3,778 (64.2%)	1,476 (62.0%)	1,044 (64.1%)	2,008 (63.3%)	2,535 (63.5%)	0.02
PVD	14,770 (86.6%)	5,111 (86.8%)	2,050 (86.2%)	1,404 (86.2%)	2,727 (86.0%)	3,478 (87.1%)	0.02
Other Cardiac	15,183 (89.0%)	5,270 (89.5%)	2,110 (88.7%)	1,440 (88.4%)	2,814 (88.8%)	3,549 (88.9%)	0.02
COPD	10,251 (60.1%)	3,552 (60.4%)	1,396 (58.7%)	995 (61.1%)	1,896 (59.8%)	2,412 (60.4%)	0.02
GI	7,958 (46.7%)	2,739 (46.5%)	1,123 (47.2%)	765 (47.0%)	1,479 (46.7%)	1,852 (46.4%)	0.01
Liver Disease	5,755 (33.7%)	1,970 (33.5%)	788 (33.1%)	558 (34.3%)	1,065 (33.6%)	1,374 (34.4%)	0.01
Dysrhythmia	14,028 (82.2%)	4,908 (83.4%)	1,945 (81.8%)	1,341 (82.3%)	2,581 (81.4%)	3,253 (81.5%)	0.03
Cancer	4,935 (28.9%)	1,683 (28.6%)	678 (28.5%)	477 (29.3%)	922 (29.1%)	1,175 (29.4%)	0.01
Diabetes	14,626 (85.8%)	5,042 (85.7%)	2,008 (84.4%)	1,408 (86.4%)	2,674 (84.4%)	3,494 (87.5%)	0.05

*: Following rows are N(%) of patients ever had comorbidity, among patients with USRDS claims

SCD, sudden cardiac death; SMD, standardized mean difference; IQR, interquartile range; CVA/TIA, cerebrovascular accident/transient ischemic attack; PVD, peripheral vascular disease; COPD, chronic obstructive pulmonary disease; GI, gastrointestinal

Supplemental Table 8. Ridge Coefficients for USRDS

	SCD	Non-SCD Cardiovascular	Infection	Others	Unknown
Age at death	0.168	0.267	0.084	0.174	0.233
Male Sex	0.121	0.105	0.112	0.159	0.038
Race/Ethnicity: Non-Hispanic Black vs. Hispanic	0.035	-0.037	0.007	0.030	-0.085
Race/Ethnicity: Non-Hispanic White vs. Hispanic	0.076	0.062	0.092	0.105	0.138
Race/Ethnicity: Others vs. Hispanic	-0.035	0.008	0.039	-0.039	-0.061
Time since ESRD incidence	0.277	0.290	0.223	0.188	0.176
Vascular Access: Fistula vs. Catheter	-0.055	-0.049	-0.137	-0.097	-0.074
Vascular Access: Graft vs. Catheter	-0.021	0.007	-0.072	0.031	-0.021
Modality: Discontinued Dialysis vs. Hemodialysis	0.354	0.399	0.397	0.506	0.708
Waitlist: On vs. Off	-0.145	-0.132	-0.085	-0.098	-0.171
Waitlist: Removed vs. Off	-0.044	-0.064	-0.013	-0.023	-0.060
Medicare: Available vs. Unavailable	0.245	0.230	0.195	0.202	0.222
Employee Group Health Plan: Available vs. Unavailable	0.038	0.008	0.025	0.012	-0.022
Number of sessions	-0.606	-0.701	-0.926	-0.755	-0.836
Prescribed body mass index (BMI)	-0.103	-0.255	-0.171	-0.162	-0.168
Pre dialysis BMI	-0.106	-0.004	-0.019	-0.086	-0.102
Post dialysis BMI	0.011	-0.015	-0.028	-0.088	-0.077
Interdialytic weight gain	-0.016	0.015	-0.045	-0.103	-0.006
Intradialytic weight loss	0.068	0.068	-0.035	0.019	0.021
Pre dialysis blood pressure, diastolic	-0.055	-0.041	-0.172	-0.016	-0.063
Pre dialysis blood pressure, systolic	-0.294	-0.276	-0.264	-0.222	-0.210
Post dialysis blood pressure, diastolic	-0.054	-0.053	-0.066	-0.070	-0.012
Post dialysis blood pressure, systolic	0.085	0.135	-0.057	0.000	0.006
Serum Urea Nitrogen Pre dialysis	-0.138	-0.040	-0.048	-0.057	-0.166
Serum Urea Nitrogen Post dialysis	0.140	0.122	0.065	0.082	0.180
Serum Ferritin	0.072	0.083	0.078	0.112	0.083
Serum Creatinine	-0.188	-0.238	-0.268	-0.126	-0.215
Serum Sodium	0.080	0.069	-0.036	0.003	0.020
Serum Potassium	0.091	0.062	0.002	0.037	0.125
Serum Chloride	-0.365	-0.289	-0.278	-0.276	-0.215
Serum CO2 Content	-0.060	-0.073	-0.025	-0.030	0.053
Serum Calcium	0.085	0.172	0.120	0.030	0.049
Serum Phosphorus	0.143	0.142	0.035	0.037	0.090
Serum Alkaline Phosphatase	0.134	0.106	0.038	0.124	0.053
Serum Glucose	0.036	0.084	0.002	-0.034	0.063
Serum Alanine Aminotransferase	0.115	0.112	0.068	0.158	0.098
Serum Total Protein	0.048	0.028	-0.011	-0.084	-0.024
Serum Albumin	-0.494	-0.401	-0.663	-0.524	-0.500
Serum Transferrin	0.106	-0.024	-0.042	0.049	-0.078
Hemoglobin	-0.141	-0.058	-0.048	-0.113	-0.065
Hematocrit	0.065	0.093	0.086	0.006	0.068
Total White Blood Cells	0.174	0.164	0.237	0.267	0.164
Platelet Count	-0.142	-0.185	-0.189	-0.158	-0.146
Serum Iron	-0.132	-0.141	-0.121	-0.104	-0.157
MCV	0.118	0.140	0.161	0.111	0.106
MCHC	-0.204	-0.208	-0.236	-0.212	-0.121

Percent Saturation	-0.060	-0.019	-0.056	-0.077	-0.025
Serum Total iron binding capacity	-0.202	-0.135	-0.139	-0.172	-0.107
Ever Arteriosclerotic heart disease: Yes vs No	-0.020	0.115	-0.179	-0.085	-0.070
Ever Arteriosclerotic heart disease: Missing vs No	-0.010	-0.016	-0.009	-0.013	-0.019
Ever Congestive heart failure: Yes vs No	-0.019	0.018	-0.005	-0.069	0.032
Ever Congestive heart failure: Missing vs No	-0.010	-0.014	-0.009	-0.014	-0.018
Ever cerebral vascular accident/Transient Ischemic Attack: Yes vs No	-0.120	0.014	-0.111	-0.087	-0.119
Ever cerebral vascular accident/Transient Ischemic Attack: Missing vs No	-0.010	-0.014	-0.010	-0.014	-0.018
Ever Peripheral vascular disease: Yes vs No	-0.227	-0.268	0.000	-0.176	-0.185
Ever Peripheral vascular disease: Missing vs No	-0.013	-0.016	-0.009	-0.016	-0.020
Ever Other Cardiac disease: Yes vs No	-0.185	-0.121	-0.041	-0.075	-0.105
Ever Other Cardiac disease: Missing vs No	-0.016	-0.018	-0.010	-0.017	-0.021
Ever Chronic obstructive pulmonary disease: Yes vs No	-0.055	-0.066	-0.076	-0.006	-0.034
Ever Chronic obstructive pulmonary disease: Missing vs No	-0.017	-0.019	-0.010	-0.017	-0.022
Ever GI: Yes vs No	-0.166	-0.201	-0.124	-0.096	-0.105
Ever GI: Missing vs No	-0.017	-0.019	-0.011	-0.017	-0.022
Ever Liver Disease: Yes vs No	-0.143	-0.193	-0.039	-0.115	-0.150
Ever Liver Disease: Missing vs No	-0.018	-0.020	-0.011	-0.017	-0.022
Ever Dysrhythmia: Yes vs No	0.077	-0.051	0.026	-0.062	-0.062
Ever Dysrhythmia: Missing vs No	-0.016	-0.021	-0.010	-0.018	-0.022
Ever Cancer: Yes vs No	-0.164	-0.190	-0.135	0.048	-0.124
Ever Cancer: Missing vs No	-0.016	-0.021	-0.010	-0.018	-0.022
Ever Diabetes: Yes vs No	-0.007	-0.017	-0.024	-0.003	-0.050
Ever Diabetes: Missing vs No	-0.016	-0.021	-0.011	-0.017	-0.023

All variables scaled to have unit variance

Supplemental Table 9. Ridge Coefficients for NDI

	SCD	Non-SCD Cardiovascular	Infection	Others	Unknown
Age at death	0.133	0.333	0.170	0.130	0.237
Male Sex	0.175	0.036	0.170	0.088	0.084
Race/Ethnicity: Non-Hispanic Black vs. Hispanic	-0.003	0.084	0.108	0.110	-0.115
Race/Ethnicity: Non-Hispanic White vs. Hispanic	0.014	0.188	0.059	0.131	0.055
Race/Ethnicity: Others vs. Hispanic	-0.056	-0.059	-0.014	-0.012	-0.003
Time since ESRD incidence	0.413	0.419	0.273	0.305	0.164
Vascular Access: Fistula vs. Catheter	-0.118	-0.074	-0.149	-0.164	-0.096
Vascular Access: Graft vs. Catheter	-0.067	-0.057	-0.114	-0.035	-0.025
Modality: Discontinued Dialysis vs. Hemodialysis	0.268	0.346	0.303	0.421	0.617
Waitlist: On vs. Off	-0.137	-0.215	-0.076	-0.093	-0.187
Waitlist: Removed vs. Off	-0.070	-0.022	0.003	-0.033	-0.042
Medicare: Available vs. Unavailable	0.378	0.347	0.272	0.314	0.296
Employee Group Health Plan: Available vs. Unavailable	0.085	0.033	-0.090	0.053	-0.071
Number of sessions	-0.577	-0.667	-0.943	-0.682	-0.778
Prescribed body mass index (BMI)	-0.175	-0.138	-0.138	-0.134	-0.094
Pre dialysis BMI	-0.017	-0.078	-0.063	-0.123	-0.172
Post dialysis BMI	-0.044	-0.018	-0.005	-0.044	-0.052
Interdialytic weight gain	-0.026	-0.085	-0.100	-0.009	0.017
Intradialytic weight loss	0.088	0.090	-0.005	-0.053	-0.002
Pre dialysis blood pressure, diastolic	-0.059	-0.048	-0.269	-0.106	-0.053
Pre dialysis blood pressure, systolic	-0.202	-0.267	-0.380	-0.230	-0.215
Post dialysis blood pressure, diastolic	-0.023	-0.072	0.116	0.013	0.046
Post dialysis blood pressure, systolic	0.000	0.122	-0.007	-0.007	-0.006
Serum Urea Nitrogen Pre dialysis	-0.071	-0.041	-0.026	-0.148	-0.161
Serum Urea Nitrogen Post dialysis	0.094	0.093	0.101	0.056	0.240
Serum Ferritin	0.033	-0.009	0.108	0.145	0.081
Serum Creatinine	-0.265	-0.226	-0.301	-0.099	-0.254
Serum Sodium	0.048	0.084	-0.013	0.040	0.068
Serum Potassium	0.049	0.072	0.010	0.176	0.042
Serum Chloride	-0.377	-0.341	-0.282	-0.374	-0.351
Serum CO2 Content	-0.076	0.002	-0.075	-0.044	-0.031
Serum Calcium	0.107	0.098	0.096	0.024	0.037
Serum Phosphorus	0.098	0.074	-0.073	0.031	0.094
Serum Alkaline Phosphatase	0.127	0.054	0.210	0.190	0.165
Serum Glucose	0.029	0.000	-0.057	-0.073	0.049
Serum Alanine Aminotransferase	0.111	0.151	0.125	0.176	-0.015
Serum Total Protein	-0.011	0.037	0.027	-0.132	-0.050
Serum Albumin	-0.445	-0.298	-0.638	-0.413	-0.467
Serum Transferrin	0.134	0.080	0.007	0.094	-0.080
Hemoglobin	-0.137	-0.148	-0.020	-0.157	-0.060
Hematocrit	-0.013	0.117	0.038	-0.028	0.042
Total White Blood Cells	0.221	0.118	0.241	0.266	0.234
Platelet Count	-0.147	-0.193	-0.182	-0.155	-0.180
Serum Iron	-0.030	-0.120	-0.068	-0.051	-0.071
MCV	0.118	0.047	0.214	0.120	0.175
MCHC	-0.223	-0.283	-0.164	-0.178	-0.085
Percent Saturation	-0.184	-0.082	-0.007	-0.186	-0.168
Serum total iron binding capacity	-0.461	-0.439	-0.410	-0.518	-0.414
Ever Arteriosclerotic heart disease: Yes vs No	-0.045	0.062	-0.225	-0.072	-0.136
Ever Arteriosclerotic heart disease: Missing vs No	-0.019	-0.027	-0.031	-0.023	-0.033

Ever Congestive heart failure: Yes vs No	-0.025	-0.030	-0.016	-0.048	-0.012
Ever Congestive heart failure: Missing vs No	-0.018	-0.027	-0.030	-0.022	-0.033
Ever cerebral vascular accident/Transient Ischemic Attack: Yes vs No	-0.169	-0.003	-0.076	-0.129	-0.198
Ever cerebral vascular accident/Transient Ischemic Attack: Missing vs No	-0.019	-0.027	-0.030	-0.023	-0.033
Ever Peripheral vascular disease: Yes vs No	-0.369	-0.432	-0.153	-0.302	-0.254
Ever Peripheral vascular disease: Missing vs No	-0.021	-0.030	-0.031	-0.024	-0.036
Ever Other Cardiac disease: Yes vs No	-0.204	-0.137	-0.236	-0.100	-0.096
Ever Other Cardiac disease: Missing vs No	-0.023	-0.032	-0.033	-0.025	-0.037
Ever Chronic obstructive pulmonary disease: Yes vs No	-0.112	-0.189	-0.098	-0.031	-0.006
Ever Chronic obstructive pulmonary disease: Missing vs No	-0.023	-0.032	-0.034	-0.024	-0.036
Ever GI: Yes vs No	-0.267	-0.183	-0.252	-0.151	-0.124
Ever GI: Missing vs No	-0.024	-0.033	-0.035	-0.025	-0.036
Ever Liver Disease: Yes vs No	-0.267	-0.246	-0.175	-0.221	-0.194
Ever Liver Disease: Missing vs No	-0.025	-0.033	-0.035	-0.025	-0.036
Ever Dysrhythmia: Yes vs No	0.051	-0.102	0.011	-0.255	-0.021
Ever Dysrhythmia: Missing vs No	-0.025	-0.034	-0.035	-0.027	-0.036
Ever Cancer: Yes vs No	-0.251	-0.294	-0.223	0.066	-0.149
Ever Cancer: Missing vs No	-0.025	-0.035	-0.035	-0.027	-0.036
Ever Diabetes: Yes vs No	-0.090	0.006	-0.020	-0.100	-0.081
Ever Diabetes: Missing vs No	-0.025	-0.034	-0.035	-0.028	-0.036

Supplemental Figure 1. One versus Rest Area Under the Receiver Operating Curve (AUC) for National Death Index (NDI) Cause of Death.

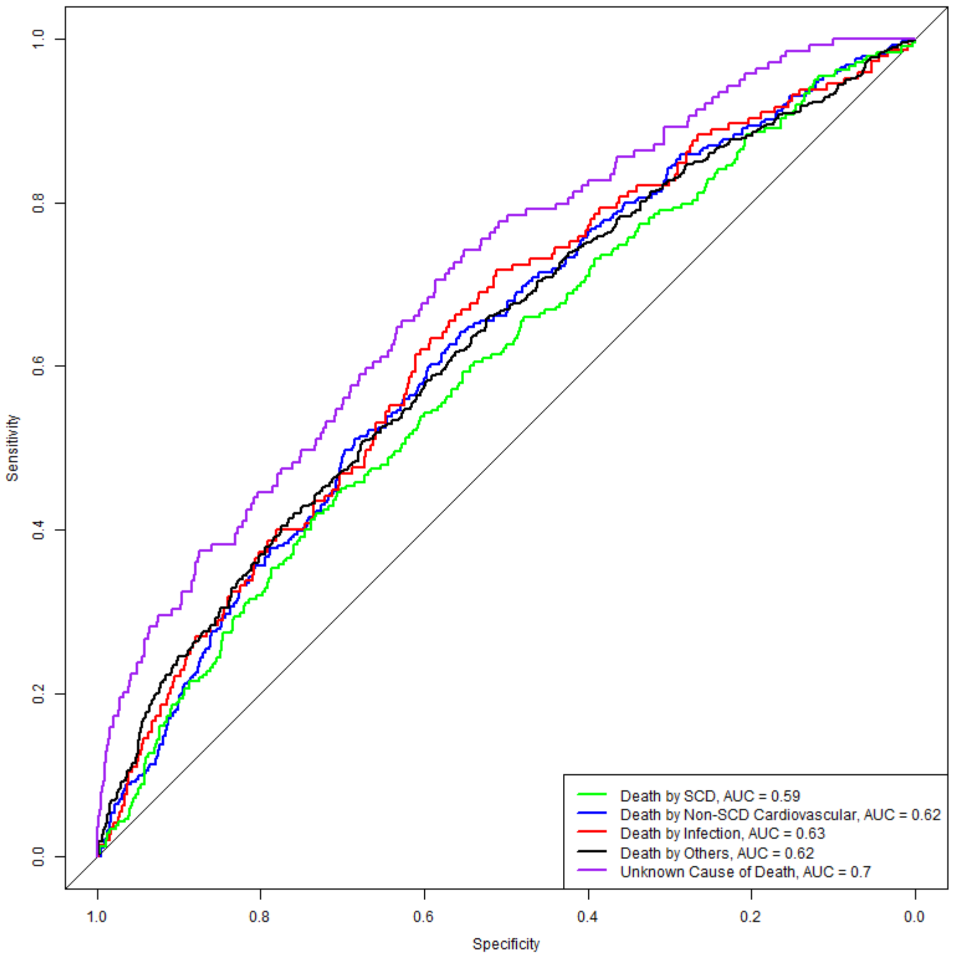

Supplemental Figure 2. Ridge Coefficients for National Death Index (NDI) Cause of Death Using Nested Case-Control Binary Outcome Models.

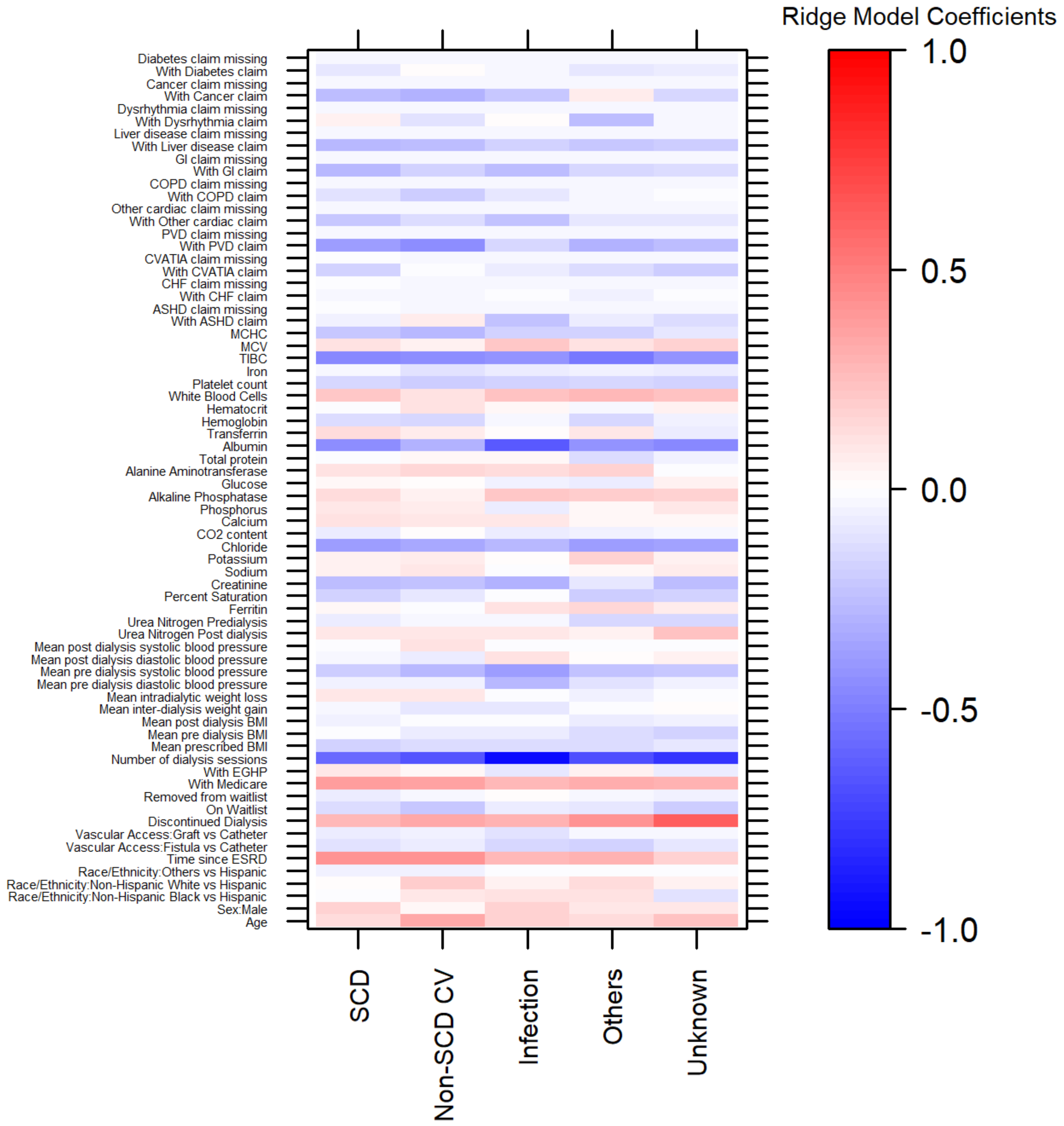

Supplemental Figure 3. Correlation Plot for Ridge Coefficients for National Death Index (NDI) Cause of Death Using Nested Case-Control Binary Outcome Models.

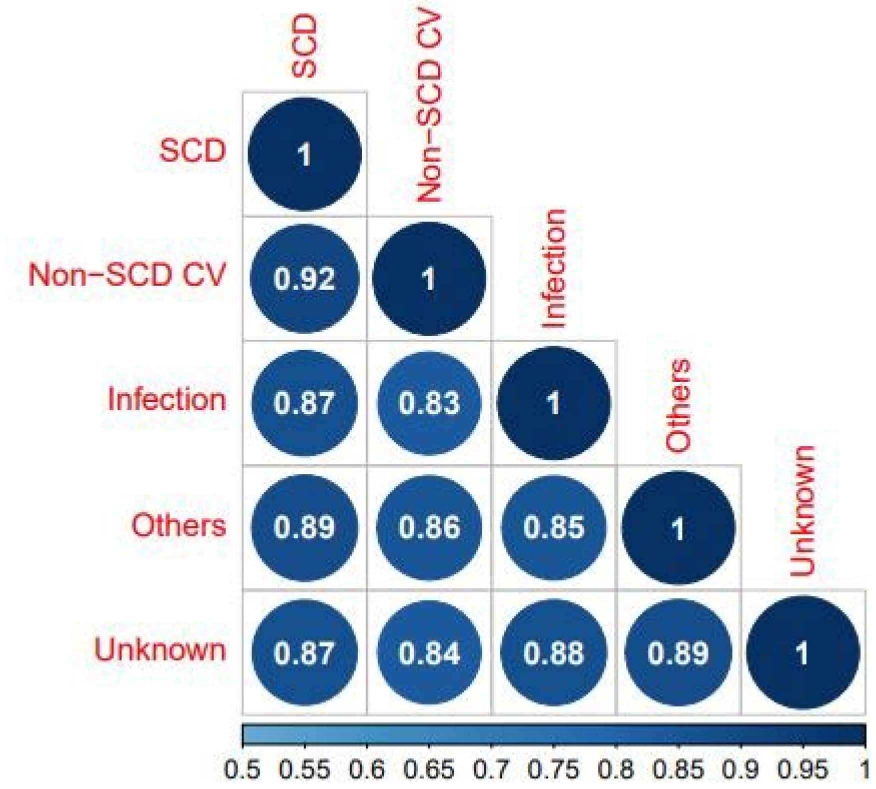

Supplement: SUPPLEMENTARY MATERIAL [file kidney360-6-432-s002.pdf]
